# Assessment of small in-frame indels and C-terminal nonsense variants of BRCA1 using a validated functional assay

**DOI:** 10.1038/s41598-022-20500-4

**Published:** 2022-09-28

**Authors:** Thales C. Nepomuceno, Ana P. P. dos Santos, Vanessa C. Fernandes, Anna B. R. Elias, Thiago T. Gomes, Guilherme Suarez-Kurtz, Edwin S. Iversen, Fergus J. Couch, Alvaro N. A. Monteiro, Marcelo A. Carvalho

**Affiliations:** 1 Divisão de Pesquisa Clínica, Instituto Nacional de Câncer, Rio de Janeiro, 20230-130 Brazil; 2 Cancer Epidemiology Program, H. Lee Moffitt Cancer Center and Research Institute, 12902 Magnolia Drive, Tampa, FL 33612 USA; 3 Department of Statistical Science, Duke University, Durham, NC 27708 USA; 4 Mayo Clinic, Rochester, MN 55905 USA; 5 Laboratório de Genética Molecular, Instituto Federal Do Rio de Janeiro, Rua Senador Furtado, Campus Rio de Janeiro121, Rio de Janeiro, RJ 20270–021 Brazil

**Keywords:** Cancer, Genetics

## Abstract

*BRCA1* (Breast Cancer 1, early onset) is linked to breast and ovarian cancer predisposition. Still, the risks conferred by a significant portion of *BRCA1* variants identified in the population remains unknown. Most of these variants of uncertain significance are missense alterations. However, the functional implications of small in-frame deletions and/or insertions (indels) are also difficult to predict. Our group has previously evaluated the functional impact of 347 missense variants using an extensively validated transcriptional activity assay. Here we show a systematic assessment of 30 naturally occurring in-frame indels located at the C-terminal region of BRCA1. We identified positions sensitive and tolerant to alterations, expanding the knowledge of structural determinants of BRCA1 function. We further designed and assessed the impact of four single codon deletions in the tBRCT linker region and six nonsense variants at the C-terminus end of BRCA1. Amino acid substitutions, deletions or insertions in the disordered region do not significantly impact activity and are not likely to constitute pathogenic alleles. On the other hand, a sizeable fraction of in-frame indels at the BRCT domain significantly impact function. We then use a Bayesian integrative statistical model to derive the probability of pathogenicity for each variant. Our data highlights the importance of assessing the impact of small in-frame indels in *BRCA1* to improve risk assessment and clinical decisions for carriers.

## Introduction

BRCA1 is a tumor suppressor involved in genome integrity maintenance acting as a platform to recruit several proteins involved in the DNA damage response to double-strand breaks (DSB)^1–5^. It also plays a central role in other biological processes such as chromatin remodeling, centrosome duplication, and transcription regulation^6–9^.

For women harboring monoallelic variants that encode dysfunctional BRCA1 protein the cumulative risk for breast or ovarian cancer by the age of 80 years is 72% and 44%, respectively^10–12^. The accurate classification of a *BRCA1* variant as pathogenic or benign is crucial to risk stratification, to establish preventive clinical options for carriers and therapeutic options for patients^13,14^.

Variants for which a premature termination of the protein, such as those resulting in nonsense and frameshift changes, can be inferred to lead to loss of function and are therefore considered pathogenic^15^. However, the large number of *BRCA1* variants of uncertain clinical significance, mostly missense and splicing variants, is an obstacle to the implementation of precision medicine^16,17^. When information is available, a multifactorial model can be used to infer the likelihood of pathogenicity based on segregation data and family or personal history^18,19^. In addition, because carrying two germline pathogenic (loss of function) alleles of *BRCA1* in embryonic lethal, a VUS that co-occurs with a known pathogenic variant is extremely likely to be benign^20^. Therefore, co-occurrence with a pathogenic variant is also considered in the multifactorial model^18^. However, the low allele frequency (< 1/10,000) of these variants in the general population impedes family and population-based analysis to determine their association with cancer risk^15^. In this context, the functional assessment of variants of uncertain significance (VUS) is a powerful tool to empirically interrogate the impact of a single variation on a specific disease-associated protein function when its loss of function cannot be inferred from changes in the genetic code or in the absence of population or clinical information^17,21–23^.

A recent comprehensive study evaluated the specificity and sensitivity of 131 published functional assays for *BRCA1* using a gold standard reference panel and validated (≥ 80% specificity and sensitivity) 22 assays^21^. Only six assays achieved benchmarks recommended by ACMG/AMP (American College of Medical Genetics & Genomics/ Association of Molecular Pathologists) to generate the strongest recommendation criteria, PS3 and BS3, for pathogenic and benign variants, respectively. Here, we use one of these assays, the transcription activation assay, to assess the function of 30 small in-frame indels located at the BRCA1 C-terminal region and derive the likelihood of pathogenicity given the functional data. Most of the data available on BRCA1 is derived from the functional impact of missense substitutions and noncanonical splicing site variants, which constitute the majority of VUS^21,24^. The consequence of small in-frame insertions and/or deletions (indels) on BRCA1 functions remains poorly understood.

These 30 variants were identified in the BRCA exchange platform and encode different protein products, such as multiple-nucleotide changes resulting in missense substitutions, deletions, and/or insertions^25^. We also evaluated the impact of a set of additional variants, composed of four single codon deletions in the linker region of the tandem BRCTs and six C-terminal nonsense variants. The data presented provides a functional assessment of classes of variants that have not been systematically explored.

## Results

### Variants selection rationale

We interrogated the functional impact of 30 in-frame indels located at the C-terminal region (aa 1396–1863) of BRCA1. Naturally occurring variants previously recorded in the population were identified in the BRCA exchange database (Table 1) (https://brcaexchange.org/). Variants were divided into three groups based on the protein outcome: (1) in-frame insertions and deletions (multiple-nucleotide changes) that result in missense substitutions; (2) small in-frame deletions (SD) that result in the deletion of an amino acid residue; and (3) small in-frame insertions (SI) that result in amino acid residue insertions. These classes of variants have not been systematically evaluated by any functional assay. Twelve additional in-frame deletion and nonsense variants were also evaluated to probe into the BRCT linker region and the extreme C-terminus (Table 2).

**Table 1:** BRCA1 small in-frame indels reported in BRCA Exchange.

Nucleotide variant^a^	HGVS protein^b^	VarCall ^c^	ACMG evidence criteria	Localization
c.4211_4212insTCAGAAGGA	p.(Leu1404_Ile1405insGlnLysGlu)	fClass 3	n.a	CC
c.4377_4379del	p.(Lys1459_Ser1460delinsAsn)	fClass 1	BS3	DR
c.4441_4442delinsCT	p.(Ala1481Leu)	fClass 1	BS3	DR
c.4510_4512del	p.(Leu1504del)	fClass 2	BS3	DR
c.4579_4580delinsAT	p.(Glu1527Met)	fClass 1	BS3	DR
c.4614_4615delinsTT	p.(Gln1538His)	fClass 1	BS3	DR
c.4676_4678delAGG	p.(Glu1559del)	fClass 1	BS3	DR
c.4771_4772delinsAA	p.(Gly1591Asn)	fClass 1	BS3	DR
c.4945_4947delinsTTT	p.(Arg1649Phe)	fClass 1	BS3	DR
c.4981_4983del	p.(Glu1661del)	fClass 5	PS3	BRCT1
c.5017_5019del	p.(His1673del)	fClass 5	PS3	BRCT1
c.5078_5080del	p.(Ala1693del)	fClass 5	PS3	BRCT1
c.5092_5093delinsAG	p.(Glu1698Arg)	fClass 2	BS3	BRCT1
c.5146_5148delinsAAA	p.(Tyr1716Lys)	fClass 5	PS3	BRCT1
c.5161_5163del	p.(Gln1721del)	fClass 5	PS3	BRCT1
c.5181_5183del	p.(Met1728del)	fClass 5	PS3	BRCT1
c.5197_5199del	p.(Asp1733del)	fClass 5	PS3	BRCT1
c.5213_5215del	p.(Gly1738del)	fClass 5	PS3	Linker
c.5218_5220delinsAAC	p.(Val1740Asn)	fClass 1	BS3	Linker
c.5219_5224del	p.(Val1740_Asn1742delinsAsp)	fClass 5	PS3	Linker
c.5234_5235delinsGG	p.(Asn1745Arg)	fClass 1	BS3	Linker
c.5238_5240del	p.(His1746del)	fClass 2	BS3	Linker
c.5275_5276delinsTG	p.(Lys1759Trp)	fClass 1	BS3	Linker
c.5321_5322delinsGG	p.(Asn1774Arg)	fClass 1	BS3	BRCT2
c.5359_5363delinsAGTGA	p.(Cys1787_Gly1788delinsSerAsp)	fClass 5	PS3	BRCT2
c.5425_5430del	p.(Val1809_Val1810del)	fClass 5	PS3	BRCT2
c.5529_5530delinsCA	p.(Leu1844Ile)	fClass 1	BS3	BRCT2
c.5565_5573del	p.(Gln1857_Pro1859del)	fClass 2	BS3	BRCT2
c.5580_5581insCCCCCCCCC	p.(His1860_Ser1861insProProPro)	fClass 1	BS3	BRCT2
c.5581_5582insCCCCCCCCA	p.(His1860_Ser1861insThrProPro)	fClass 1	BS3	BRCT2

^a^NM_007294.3.

^b^NP_009225.1.

^c^Varcall assessment of pathogenicity. Functional Classes: fClass 1, non-pathogenic; fClass 2, likely not pathogenic; fClass 3, uncertain; fClass 4, likely pathogenic; and fClass 5, pathogenic).

CC: Coiled-coil motif.

DR: Disordered region.

n.a., not applicable.

**Table 2:** BRCA1 small in-frame deletions and nonsense variants in the linker region and the extreme C-terminus.

Nucleotide variant^a^	HGVS protein^b^	VarCall^c^	ACMG evidence criteria	Localization
c.5233_5235del	p.(Asn1745del)	fClass 1	BS3	Linker
c.5269_5271del	p.(Asp1757del)	fClass 1	BS3	Linker
c.5272_5274del	p.(Arg1758del)	fClass 1	BS3	Linker
c.5275_5277del	p.(Lys1759del)	fClass 1	BS3	Linker
c.5566_5568delCCCinsTGA	p.(Pro1856Ter)	fClass 5	PS3	BRCT2
**c.5569C > T**	**p.(Gln1857Ter)**	fClass 2	BS3	BRCT2
c.5572_5574delATCinsTGA	p.(Ile1858Ter)	fClass 2	BS3	BRCT2
c.5575_5577delCCCinsTGA	p.(Pro1859Ter)	fClass 2	BS3	BRCT2
c.5578_5580delCACinsTGA	p.(His1860Ter)	fClass 2	BS3	BRCT2
c.5581_5583delAGCinsTGA	p.(Ser1861Ter)	fClass 2	BS3	BRCT2
c.5584_5586delCACinsTGA	p.(His1862Ter)	fClass 2	BS3	BRCT2
c.5588_5589delACinsGA	p.(Tyr1863Ter)	fClass 2	BS3	BRCT2

^a^NM_007294.3.

^b^NP_009225.1.

^c^Varcall functional Classes: fClass 1, non-pathogenic; fClass 2, likely not pathogenic; fClass 3, uncertain; fClass 4, likely pathogenic; and fClass 5, pathogenic).

Variant in bold is the only one reported in BRCA Exchange.

### In-frame indels resulting in missense substitutions

**Figure 1:**
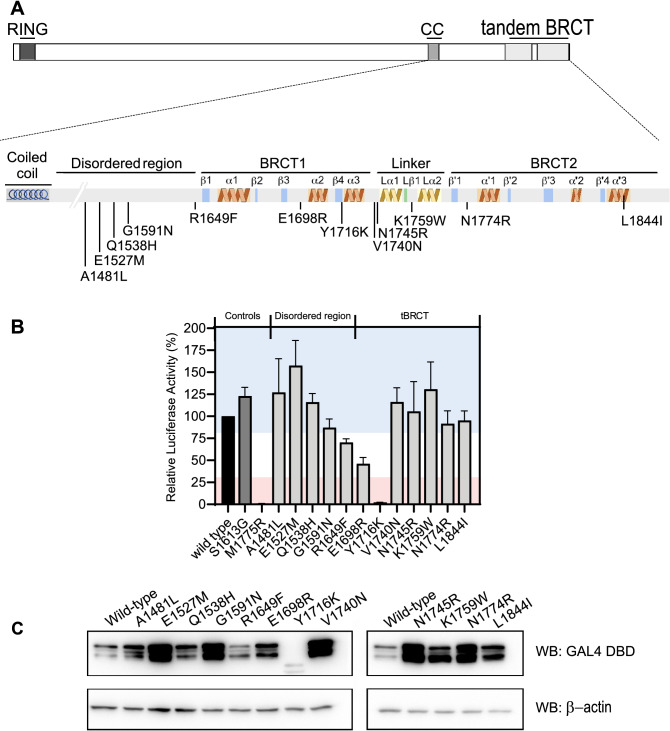
Transcription activation assay for indels that result in missense substitutions. (**A**) Schematic representation of BRCA1, with its domains. CC: Coiled-coil motif. The studied region (aa 1396–1863) is enlarged with schematic representation of the secondary structures and connecting loops in the BRCT domains. (**B**) Transcription activity of indels that result in missense substitutions (light gray bars) shown as a percentage of wild type activity (± Standard error of three independent experiments). The wild type control is represented with a black bar. The known benign p.(S1613G) variant is represented with a dark gray bar. Light red and blue backgrounds represent values below 30% and above 80%, respectively. (**C**) GAL4 DBD-BRCA1 protein levels in HEK293FT cells. Immunoblot using anti-GAL4DBD and anti-β-actin.

Our dataset is composed of 12 variants leading to missense substitutions, p.(A1481L), p.(E1527M), p.(Q1538H), p.(G1591N), p.(R1649F), p.(E1698R), p.(Y1716K), p.(V1740N), p.(N1745R), p.(K1759W), p.(N1774R), and p.(L1844I), located throughout the C-terminal region. Five of them, p.(A1481L), p.(E1527M), p.(Q1538H), p.(G1591N), and p.(R1649F) are located at the disordered region between the coiled-coil motif and the tBRCT domain (Fig. 1A). In line with previous observations, variants situated outside of the tBRCT have modest impact on BRCA1 TA function (Fig. 1B)^26,27^. The p.(R1649F) variant located close to the N-terminal border of the BRCT1 (S1651), showed a modest reduction in transcription (~ 30%). Similar results were previously observed for single nucleotide variants (SNV) leading to missense changes at the 1649 position^27^.

The tBRCT p.(E1698R) and p.(Y1716K) variants resulted in functional impact, presenting 46% and 3% of wild-type activity, respectively (Fig. 1B). The p.(E1698R) is located between BRCT1 β3 and α2, and the p.(Y1716K) is located at the end of β4 secondary structures (Fig. 1A). The remaining tBRCT variants, p.(V1740N), p.(N1745R), p.(K1759W), p.(N1774R), and p.(L1844I) had no significant impact on the TA function (less than 20% reduction) (Fig. 1B). Variant p.(Y1716K) protein product showed an altered migration pattern and a markedly reduced level observed in immunoblotting analysis, suggesting that its loss of activity is due to protein instability (Fig. 1C).

### Small in-frame deletions leading to amino acid residue deletions

**Figure 2:**
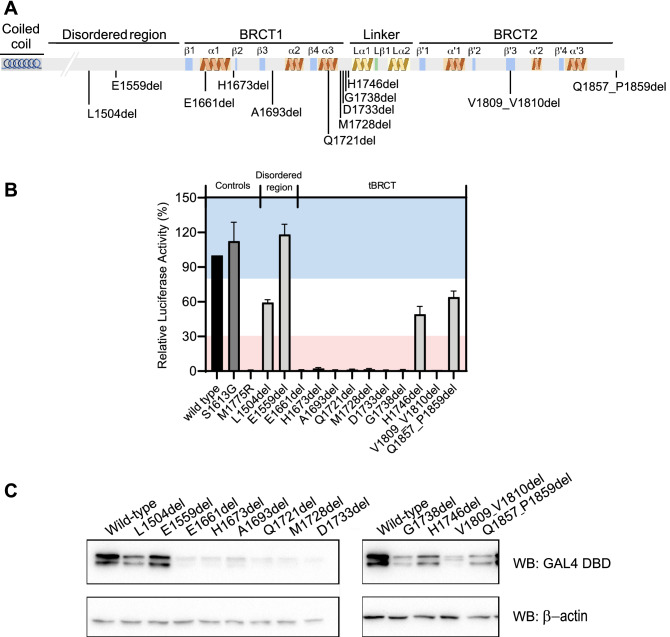
Transcription activation assay for small in-frame deletions that result in amino acid residue deletions. (**A**) schematic representation of the studied region (aa 1396–1863) depicting the secondary structures and connecting loops in the BRCT domains. (**B**) Transcription activity of small in-frame deletions (light gray bars) shown as a percentage of wild type activity (± Standard error of three independent experiments). The wild type control is represented with a black bar. The known benign p.(S1613G) variant is represented with a dark gray bar. Light red and blue backgrounds represent values below 30% and above 80%, respectively. (**C**) GAL4 DBD-BRCA1 protein levels in HEK293FT cells. Immunoblot using anti-GAL4DBD and anti-β-actin.

We tested 12 variants with small in-frame deletions leading to amino acid residue deletions, ten located at the tBRCT and two at the disordered region (Fig. 2A). Interestingly, the deletion of the p.(L1504del) residue at the disordered region resulted in reduced protein abundance and in activity approximately 35% lower than the wild type BRCA1 activity (Fig. 2B,C). The other variant located at the disordered region, p.(E1559del) did not impact the BRCA1 protein expression level nor its activity (Fig. 2B,C).

Most of the single amino acid residue deletions within the tBRCT resulted in the abrogation of TA and a marked reduction of protein levels (Fig. 2B,C). Further, we also observed that the loss of three amino acid residues at the C-terminal border of the tBRCT in p.(Q1857_P1859del) variant had a modest impact on TA (~ 30% reduction). This was not the case for p.(V1809_V1810del) which had a dramatic effect on function (Fig. 2B).

Collectively, these data show that in-frame deletions at the linker and the C-terminal border had a less prominent impact on the TA function than at other sites on BRCA1 tBRCT, suggesting that specific regions in the domain are more tolerant to single amino acid residue loss.

### Small in-frame insertions (SI)

**Figure 3:**
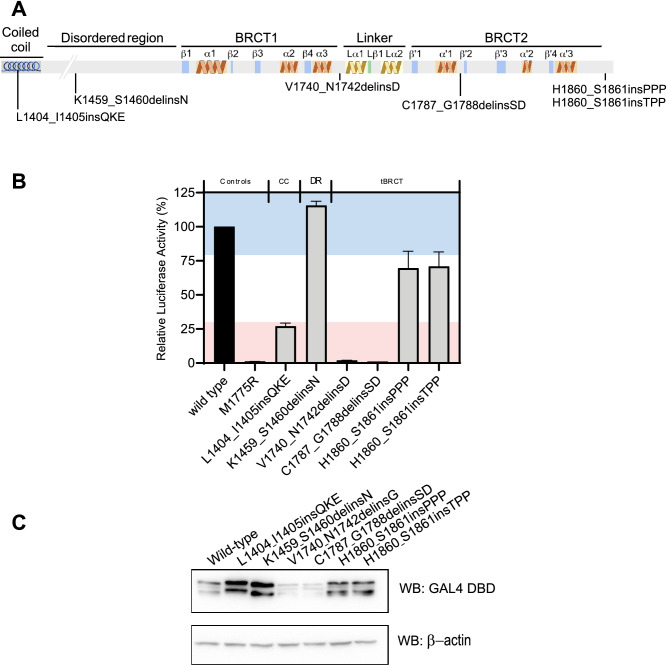
Transcription activation assay for indels that result in amino acid residue insertions. (**A**) schematic representation of the studied region (aa 1396–1863) depicting the secondary structures and connecting loops in the BRCT domains. (**B**) Transcriptional activity of small in-frame deletions (light gray bars) shown as a percentage of wild type activity (± Standard error of three independent experiments). The wild type control is represented with a black bar. The known loss-of-function M1775R variant is represented in red. Light red and blue backgrounds represent values below 50% and above 80%, respectively. Regions enclosing the variants are depicted on the top of the bar graph. CC: Coiled-coil. DR: Disordered region. (**C**) GAL4 DBD-BRCA1 protein levels in HEK293FT cells. Immunoblot using anti-GAL4DBD and anti-β-actin.

We also assessed the functional impact of six SI variants leading to the insertion of amino acid residues in the C-terminal portion of BRCA1. Interestingly, the p.(L1404_I1405insQKE) variant located at the coiled-coil presented loss of function with a ~ 70% reduction in TA relative to wild type BRCA1 (Fig. 3A,B). The p.(K1459_S1460delinsN) variant, which is located at the disordered region, displayed activity compared to the wild type BRCA1. On the other hand, the p.(V1740_N1742delinsD) and p.(C1787_G1788delinsSD) variants located at the tBRCT domain resulted in loss of function, displaying reduced activity and protein levels (Fig. 3A–C). In contrast, small insertions of three residues at the C-terminus, p.(H1860_S1861insPPP) and p.(H1860_S1861insTPP), had a modest impact on activity (~ 30% reduction) and presented normal protein levels (Fig. 3B,C). These observations show that the tBRCT domain is sensitive to small in-frame indels and that the C-terminal end of BRCA1 could be more SI tolerant.

### Single codon deletions at the Linker

**Figure 4:**
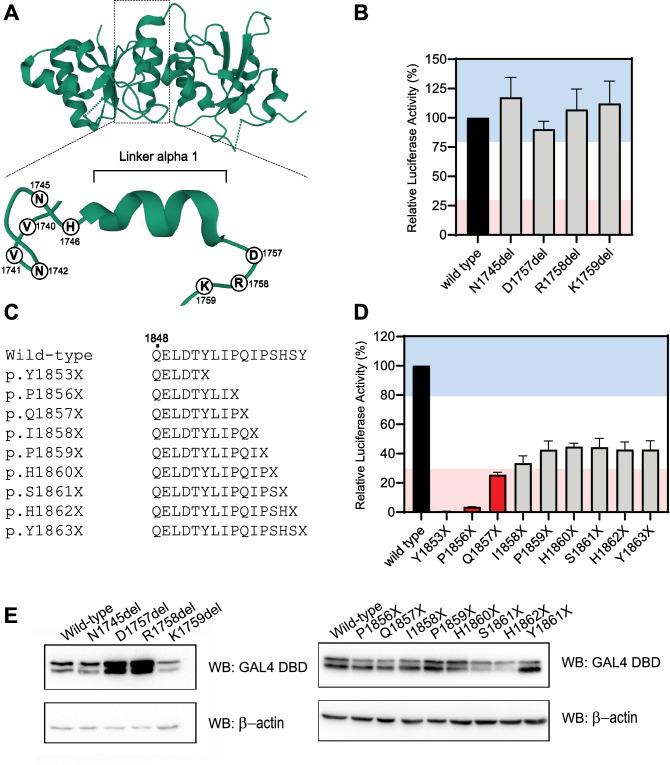
Transcription activation assays for single codon deletions at the linker region and nonsense variants at the C-terminus border of BRCA1. (**A**) structure of BRCA1 tandem BRCT domains (PDB 1JNX). The linker region and the C-terminal border are depicted with dashed boxes. The linker is rotated (90°) and enlarged to show amino acid residue positions tested. (**B**) Transcriptional activity of single codon deletions located at the linker region between BRCT domains (light gray bars) shown as a percentage of wild type activity (± Standard error of three independent experiments). The wild type control is presented with a black bar. (**C**) BRCA1 C-terminal end variants alignment (**D**) Transcriptional activity of nonsense variants located at the C-terminus of BRCA1 (light gray bars). The wild type control is represented with a black bar. Previously evaluated variants are represented with red bars. Light red and blue backgrounds represent values below 30% and above 80%, respectively. (**E**) GAL4 DBD-BRCA1 protein levels in HEK293FT cells. Immunoblot using anti-GAL4DBD and anti-β-actin.

The H1746 residue is located next to the first α-helix in the linker at the tBRCT (Fig. 2A) and is sensitive to missense substitutions^27^. We observed that the deletion of the H1746 residue had less impact than other deletions at the BRCT1 or BRCT2 regions (Fig. 2B). We hypothesized that single amino acid deletions in non-structured portions of the linker would not impact the BRCA1 TA function. To address this question, we tested the impact of the individual loss of four other residues (N1745, D1757, R1758, and K1759) located between secondary structures in the linker region (Table 2) (Fig. 4A). Interestingly, all deletions displayed transcriptional activity ≥ 80% of the wild type (Fig. 4B). Different from what was observed for variants in the BRCT1 or BRCT2, none of these variants had a negative impact on protein levels (Fig. 4C). This could be indicative that residues located outside of secondary structures at the linker region are more tolerant to small in-frame deletions than others in the BRCT structured domains.

### Nonsense C-terminal variants

The observation that in-frame deletions (Fig. 2B) or in-frame insertions (Fig. 3B) at the C-terminal border of the tBRCT had a modest effect on the BRCA1 transcriptional activity led us to test the impact of additional nonsense variants in the region. To understand the importance of the last BRCA1 amino acid residues in maintaining the tBRCT functional integrity we generated, in addition to the previously examined p.(Q1857X), other six nonsense variants, p.(I1858X), p.(P1859X), p.(H1860X), p.(S1861X), p.(H1862X), and p.(Y1863X) that resulted in the deletion of one to six amino acid residues in the BRCA1 C-terminus (Fig. 4C). We also tested p.(Q1857X) and p.(P1856X) as reference nonsense variants. The loss of a single amino acid residue at the C-terminus of BRCA1 (Y1863) led to  ~ 60% reduction in activity; and the deletion of up to five residues presented similar transcriptional activity to the one observed for the p.(Y1863X) (Fig. 4D). None of the nonsense variants resulted in reduced protein expression levels (Fig. 4E). Collectively, our data illustrate the importance of the last C-terminal amino acid residues of BRCA1 in sustaining its full activity.

### Probability of pathogenicity given the functional data

To determine whether a variant’s activity in the transcriptional assay is associated with pathogenicity (i.e., increased risk of breast and ovarian cancer) we applied a computational model, VarCall, that has been extensively used in functional assays^27–29^. VarCall estimates the probability of pathogenicity of a variant given the functional data (Supplementary Table S1)^28^. We then use a “functional class” (fClass) scoring scheme based on thresholds recommended by IARC to obtain a categorical classification for the variant^30,31^: PrDel ≤ 0.001 = fClass 1 (non-pathogenic); 0.001 < PrDel ⩽0.05 = fClass 2 (likely not pathogenic); 0.05 > Pr-Del⩽0.95 = fClass 3 (uncertain); 0.95 < PrDel ⩽ 0.99 = fClass 4 (likely pathogenic); and PrDel > 0.99 = fClass 5 (pathogenic). Under the ACMG/AMP framework^32^, variants in functional classes 1 and 2 correspond to BS3 (strong support for non-pathogenicity) and variants in functional classes 4 and 5 correspond to a PS3 (strong support for pathogenicity) (Table 1).

Variants examined in the disordered region, irrespective of groups based on the protein outcome did not impair function and were classified as fClass 1 and 2 (non-pathogenic and likely non-pathogenic) (Table 1) (Supplementary Table S1). A high-resolution graph with the Variant specific effects is shown in Supplementary Fig. S1. All six amino acid deletions located in the BRCT1 while only one in six amino acid deletions in the linker region were classified as fClass 5. Two out of five variants, a deletion and a deletion and insertion of amino acids, tested in the BRCT2 domain scored as fClass 5 (Tables 1 and 2) (Supplementary Table S1). In summary, the data shows that BRCT domains are very sensitive to in-frame deletions and insertions and variants in these domains are more likely to be pathogenic, while the disordered and linker regions may better tolerate these alterations.

Application of VarCall is particularly illuminating in the case of variants that show partially reduced activity, as exemplified by variants at the extreme C-terminus (Fig. 4D). Although function was impaired by premature termination even when only one amino acid was lost in p.(Y1863X), only variant p.(Q1856X) was deemed pathogenic (Table 2).

Because VarCall considers the totality of the functional data for all variants tested, the probability of pathogenicity for each variant is recalculated after the addition of each dataset. Each new dataset adds to information about the effects distribution for the benign and pathogenic components and allows for variants that were previously a VUS (no call) to be called benign or pathogenic. After the addition of the dataset in the current study, four missense variants, p.(G1743R), p.(P1749T), p.(G1788D), and p.(L1404P) moved from a no-call range (VUS) to neutral/benign, and one variant, p.(A1708V), moved from a no-call region to pathogenic (Supplementary Figure S2).

## Discussion

The accurate classification of *BRCA1* variants has a major impact on genetic counseling of carriers and their families, and on the clinical management of patients with breast and ovarian cancer. The relative low frequency of variant alleles hinders their classification by population-based studies. Functional assays are powerful tools to aggregate information on the impact of these variations for the purposes of clinical management.

The functional impact of BRCA1 single-nucleotide substitutions that result in missense variants has been extensively studied during the last 15 years with functional data available for over 2700 variants^23,26,27,29,33–36^. Still, little is known about the consequence of small in-frame indels that can lead to different protein products on BRCA1 function. Here we assessed variants with multiple-nucleotide changes resulting in missense, amino acid deletion and insertion variants in the C-terminal portion of BRCA1 (1396–1863 amino acid residues) using the validated BRCA1 transcriptional activation assay coupled to VarCall, a computational model to estimate the probability of pathogenicity^27^.

Analysis of in-frame indels supports the notion that amino acid substitutions, deletions or insertions in the disordered region do not significantly impact activity and are not likely to constitute pathogenic alleles. This is illustrated by p.(L1504del) variant, which had the largest reduction in activity, but the magnitude of the impact was still not enough for a classification as likely pathogenic/pathogenic classification. This is in line with previous data for single-nucleotide missense changes in which none of the 95 variants tested scored as pathogenic^27^.

In-frame indel variants that result in amino acid substitutions located at the BRCT domains did not impact function suggesting that positions V1740, N1745, K1759, and L1844 are tolerant to amino acid substitutions. This is conclusion is supported by data from a saturation genome editing (SGE) study that observed tolerance to different amino acid substitutions at these residue positions^34^. For the purposes of variant classification, we have shown that normalization using protein levels may lead to inflated activity levels when an amino acid substitution or deletion leads to protein instability^21,27^. This is the case for missense variant p.(Y1716K), several in-frame deletion variants, p.(E1661del), p.(H1673del), p.(A1693del), p.(Q1721del), p.(M1728del), p.(D1733del), p.(G1738del), and p.(Q1809_1810del), and variants in which indels result in amino acid insertions, p.(V1740_N1742delinsG) and p.(C1787_G1788delinsSD). Differences in protein levels may also impact on reporter activity when a variant with reduced transactivation activity is overexpressed because higher steady state levels may compensate for the reduced activity. This is the case for indels leading to single amino acid substitution p.(E1527M), p.(G1591N), p.(V1740N), p.(N1745R), p.(K1759W), p.(N1774R), and p.(L1844I). It is unclear why these indels lead to higher steady state levels, but it may confound analysis. Of seven previously tested variants that were overexpressed in relation to the wild type and scored as no functional impact^26,27,37,38^, only p.(L1564P), p.(S1623G), p.(M1628T), and p.(M1652I) were subsequently classified as IARC Class 1 (benign), and two variants, p.(S1613C) and p.(Q1826H) are substitutions of a different amino acid in codons with previous Class 1, p.(S1613G) and Class 2, p.(Q1826L). Although the data suggest that the overexpressed variants have no impact on function, further studies are needed, and caution is warranted when interpreting pathogenicity of these variants.

On the other hand, a significant fraction (11/21) of in-frame indel variants that result in amino acid deletions and insertions at the BRCT significantly impact function. Previously, a single study in the literature assessed the functional impact of a single amino acid residue deletion in the BRCA1 tBRCT. The p.(V1688del) variant displayed in a striking reduction of the transcriptional activity^39^.

The BRCA1 tBRCT-mediated transcriptional activity is linked to its structural integrity^33^. Missense variants that disrupt secondary structures of the tBRCT abrogate BRCA1 functions^40,41^. Interestingly, although most (7/8) single amino acid deletions in the BRCT domains led to loos of function, deletion of H1746 did not abolish BRCA1 function. Five missense variants at the 1746 codon have been previously evaluated and all exhibited loss of activity, ranging from 1.66% in p.(H1746D) to 59% in p.(H1746Q), suggesting that the functional impact of amino acid substitutions can be more detrimental than the deletion of a wild type residue^27^. Residues located in loops and turns between secondary structures in the linker region are not essential for the tBRCT transcriptional activity as illustrated by the p.(N1745del).

Variants leading to alterations at the extreme C-terminus of BRCA1 present a more complex scenario for risk assessment. The variant p.(Q1857_P1859del), which leads to the loss of three residues at the C-terminus but retains the last four amino acid residues of the protein, retained ~ 65% of the wild type activity and scores as likely benign. Similarly, small in-frame insertions at or after H1860 the C-terminus of the protein did not significantly impact its function. Interestingly, premature termination at Y1863 (only a single amino acid lost) or before (1856–1862) leads to a partial reduction in activity but only p.(P1856X) led to a marked reduced activity associated with pathogenicity suggesting that this region has a critical role in maintaining the tBRCT structural organization. It is worth noting that current models of classification, which are related to clinical management options, only discriminate variants that are associated with high risk (Relative risk > 4) from those that are not associated with high risk. Therefore, some variants that have reduced activity, and may be associated with a smaller, but still elevated relative risk, that under current guidelines, may not trigger current clinical management options. Clinical assessment of these hypomorphic variants is currently an active field of study.

The choice of the transcription assay to assess these underexplored groups of variants was based on several criteria. The transcription assay has been calibrated with a large number of variants of known risk in the population, benchmarked, and extensively validated (Sensitivity = 0.98; 95%CI 0.87–1.0; Specificity = 1.0; 95%CI 0.92–1.0)^21^. In addition, the use of VarCall allows adjustment for batch effects and uses activity data of a large set of variant effects (n = 392 in the current study) to estimate the likelihood of pathogenicity. The power of VarCall is illustrated by the progressive refinement of the probabilities of pathogenicity for all variants in the dataset after the addition of new data. The addition of 641 measurements (data generated in the current study) to 5,634 previous measurements (previous studies) led to the assignment of five previously analyzed variants that had remained as VUS to benign or pathogenic classes.

The transcription assay is accepted by the ENIGMA (Evidence-based Network for the Interpretation of Germline Mutant Alleles) consortium as a source of evidence for variant classification^18^. According to the ACMG/AMP guidelines for the use of functional data for clinical variant interpretation, validated functional assays can be used as a source of evidence to classify variants according to their likelihood of pathogenicity^32^. Under the ACMG/AMP framework, the transcriptional assay generates the highest evidence strength codes possible for a functional assay: BS3 for benign and PS3 for pathogenic variants^21^.

Several lines of evidence support the role for BRCA1 in transcriptional regulation. First, the BRCA1 C-terminal can activate transcription when fused to a heterologous DNA binding domain^42–44^. Second, BRCA1 is found in complex with the RNA Polymerase II holoenzyme and can bind DNA through specific sequences^45–47^. Finally, BRCA1 is enriched at transcription start sites in a genome-wide manner^48^. Importantly, mutations that disrupt transcriptional activation are associated with the risk for breast and ovarian cancer^27^. Our approach also has limitations which include the assay inability to assess effects on mRNA transcripts and the fact that activity is measured not as a full-length protein but as a fusion of the BRCA1 C-terminal region to yeast GAL4 DBD. The extremely high concordance between results in transcription assays, viability, and homology recombination-based assays indicate that, despite limitations, the transcriptional assay is a reliable monitor of the integrity of the BRCA1 C-terminus.

In summary we provide a functional assessment and probabilities of pathogenicity for group of variants that had not been systematically evaluated, namely small in-frame indels. The impact of these variants on function cannot be directly inferred from the DNA code and current in silico prediction tools are not adequate for prediction, highlighting the importance of direct experimental assessment of activity to improving risk assessment for carriers of *BRCA1* variants.

## Methods

### Variant selection

Known *BRCA1* (NM_007294.3) (OMIM 113,705) variants were downloaded from BRCA exchange on 07/30/2019^25^. These variants have been recorded at least once in clinical or population-based studies. We considered ‘small in-frame indels ‘ genomic variants that resulted in substitution, deletion and/or insertion of up to four amino acid residues. We selected 30 small in-frame indels located within exons 13 to 24 for further functional evaluation by the transcription assay (Table 1). In addition, we designed four single-codon deletions at the tBRCT linker, p.(N1745del), p.(D1757del), p.(R1758del), and p.(K1759del) and six nonsense variants at the C-terminal border of the tBRCT domain, p.(I1858X), p.(P1859X), p.(H1860X), p.(S1861X), p.(H1862X), and p.(Y1863X) (Table 2). As a reference we also assessed two previously tested variants p.(P1856X) and p.(Q1857X).

### Cell lines

HEK293FT cells were purchased from Invitrogen and maintained in DMEM supplemented with 10% FBS at 37 °C in a 5% CO_2_ atmosphere. Cells are periodically assessed for mycoplasma contamination and authenticated by STR analysis.

### Plasmid constructs

Variants were generated by site-directed mutagenesis as previously described^27^. In brief, mutagenesis was performed in a single-PCR reaction, using the pcDNA3_GAL4DBD:BRCA1 13/24 as the template and specifically designed primers (Supplementary Table S2). Mutations were introduced in primers and PCR was conducted following PrimeStar DNA polymerase manufacture’s recommendations (Takara Bio Inc.). PCR products were subjected to *Dpn*I for template digestion, following the manufacture’s protocol (Thermo Fisher Scientific) and then transformed into the *E. coli* DH5α strain. Ampicillin-resistant clones were selected and subjected to Sanger sequencing to confirm the desired variant and the absence of additional mutations.

### Transcriptional activity (TA) assay

TA assays were performed using the previously described pcDNA3_GAL4DBD:BRCA1 13/24 construct^26,27^. The wild type (WT, GenBank® accession U14680), pathogenic p.(M1775R) and p.(Y1853X) (both IARC class 5), and benign p.(S1613G) (IARC class 1) variants were used as controls. Previously evaluated nonsense variants p.(Q1857X) and p.(P1856X) were used as reference variants in the analysis of the extreme C-terminus^27^.

BRCA1 constructs (controls or variants) were co-transfected with the pG5Luc reporter and the phGR-TK plasmids coding for the *Photinus pyralis* and *Renilla reniformis* luciferase expression, respectively. Transfections were conducted using Polyethylenimine (Polysciences Inc.) into HEK293FT cells as described previously^27^. Cells were harvested 24 h post-transfection and TA was indirectly quantified using the Dual-Luciferase Reporter Assay System (Promega).

Variants relative luciferase activities were first normalized using the internal luciferase control in which *Renilla* luciferase is driven by a constitutively active promoter and activity was expressed as percentage of the wild type. Our decision to normalize results using the internal reporter control (*Renilla* luciferase) only and not use protein levels is based on the following data. Provided that all DNA preps (including controls) to be used in each assay are prepared in parallel^49^, and independent replicates are conducted, the TA is extremely robust when normalizing each well against its own internal *Renilla* luciferase control only (Sensitivity of 0.98 [95%CI 0.87–1.0] and Specificity of 1.0 [95%CI 0.92–1.0)^21^. A direct comparison showed that both methods of normalization had similar CV distribution but normalization by internal luciferase control only displayed higher sensitivity than when protein levels are incorporated^27^. There was a 100% agreement with between the reporter only normalized calls and the recent saturation genome editing study of BRCA1 VUS^34^. Despite this analysis being limited in scope and the unavailability of reference variants in this set, it strongly suggests that the disadvantages of normalizing by protein levels outweigh its value as an internal reference. Langerud et al. independently arrived at similar conclusions^50^. This is likely due to the following technical reasons.

In mid- to high-throughput studies, luciferase assays are performed in 96-well or 384-well plates. Western blots for protein levels are normally done using parallel transfections in a larger surface area plate to obtain enough lysate (typically 6 or 12-well plates). Thus, protein levels observed are not direct measurements from each well. Protein instability is the common underlying cause of loss of function in most coding variants. Thus, while normalization using the level of variant protein expression mitigates problems due to error during transfection (*e.g*., incorrectly adding less plasmid for a specific variant to the transfection mix) it may potentially yield incorrect results for the purposes of clinical annotation.

To minimize technical errors, improve quality control, and detect when an error occurs all expression plasmids (including the wild-type, positive, and negative controls) used in each batch of transfection are prepared in the same batch of mini-preps. Concentration and 260/280 ratios are checked by nanodrop and visualized by gel electrophoresis, to guarantee that preps contain comparable amounts of supercoiled DNA. Plasmids are verified by Sanger of both strands. Transfection assays are done in 96-well plates and transfection mixes (containing both reporters and transfection reagents) are used for all wells, minimizing the effect of adding no or less reagents in individual wells. We follow an optimized protocol for transfections in reporter assays^51^. Every variant reported in this paper is tested using four technical and three independent replicates, minimizing the effects of possible plasmid swaps or other technical errors. Relative luciferase activity for all replicates can be found in Supplementary Table S3.

### Statistics

Results are presented in bar graphs of the mean percentage relative to wild type luciferase activity ± SEM of three independent experiments. To estimate the probability of pathogenicity given the functional data we used the computation method VarCall^28^ using the input dataset (Supplementary Table S4) which merged the results from the Fernandes et al.^27^ with the variants in this study. Next, we incorporated the results obtained for the 42 variants in the present study into the VarCall algorithm in a combined analysis with data from published variants in order to estimate the likelihood of pathogenicity of 390 variants^28,30^ (Supplementary Table S1). The output from VarCall represents the probability of pathogenicity given the effects on the functional capacity of the variant scheme^30^. Using the posterior probability calculation (PrDel) we generate the following functional classifications (fClass): PrDel ≤ 0.001 as fClass 1 (non-pathogenic), 0.001 < PrDel ⩽0.05 as fClass 2 (likely not pathogenic), 0.05 > Pr-Del⩽0.95 as fClass 3 (uncertain), 0.95 < PrDel ⩽ 0.99 as fClass 4 (likely pathogenic), and PrDel > 0.99 as fClass 5 (pathogenic). The model refines the pathogenicity for all variants in the set every time a new set of variants is added. Four previously tested missense variants, p.(G1743R), p.(P1749T), p.(G1788D), and p.(L1404P) moved from a no-call range (fClass 3; VUS) to neutral/benign (fClass 2), and one variant, p.(A1708V), moved from a no-call region to pathogenic (fClass 4) (Supplementary Figure S2). Codes for VarCall are available upon request.

### Immunoblotting

BRCA1 constructs were transfected into HEK293FT cells using Polyethylenimine (Polysciences Inc.) as described previously^27^. Cells were harvested 24 h post-transfection and lysed with mild-RIPA buffer (NaCl 120 mM; Tris–Cl 50 mM (pH 7.4); EDTA 1 mM (pH 8.0); Triton X-100, 1%) supplemented with protease inhibitors (Sigma Co.). Whole cellular extracts were evaluated by immunoblotting using mouse monoclonal anti-GAL4 DBD (Santa Cruz Biotechnology, catalog number sc-510) and anti-β-actin (Santa Cruz Biotechnology, catalog number sc-47778) as primary antibodies. Horseradish peroxidase-conjugated anti-mouse IgG (Santa Cruz Biotechnology, catalog number sc-516102) was used as the secondary antibody. Bio-Rad ChemiDoc MP imaging system was used for signal detection. Image acquisition was optimized for the wild-type level of expression using the high-resolution default of the Bio-Rad Image Lab software. Original, unedited western blot images are shown in Supplementary Figure S3.

## Supplementary Information

Supplementary Information 1.

Supplementary Information 2.

Supplementary Information 3.

Supplementary Information 4.

## Data Availability

The datasets generated during the current study are available in Supplementary Tables.
