# Supplementary figures and images for: Assessment of small in-frame indels and C-terminal nonsense variants of BRCA1 using a validated functional assay
Source: Sci Rep. 2022 Sep 28;12:16203. doi: 10.1038/s41598-022-20500-4 (PMC9519549; doi:10.1038/s41598-022-20500-4)

Supplementary Figure S1. Estimated variant-specific effects for 390 BRCA1 variants.

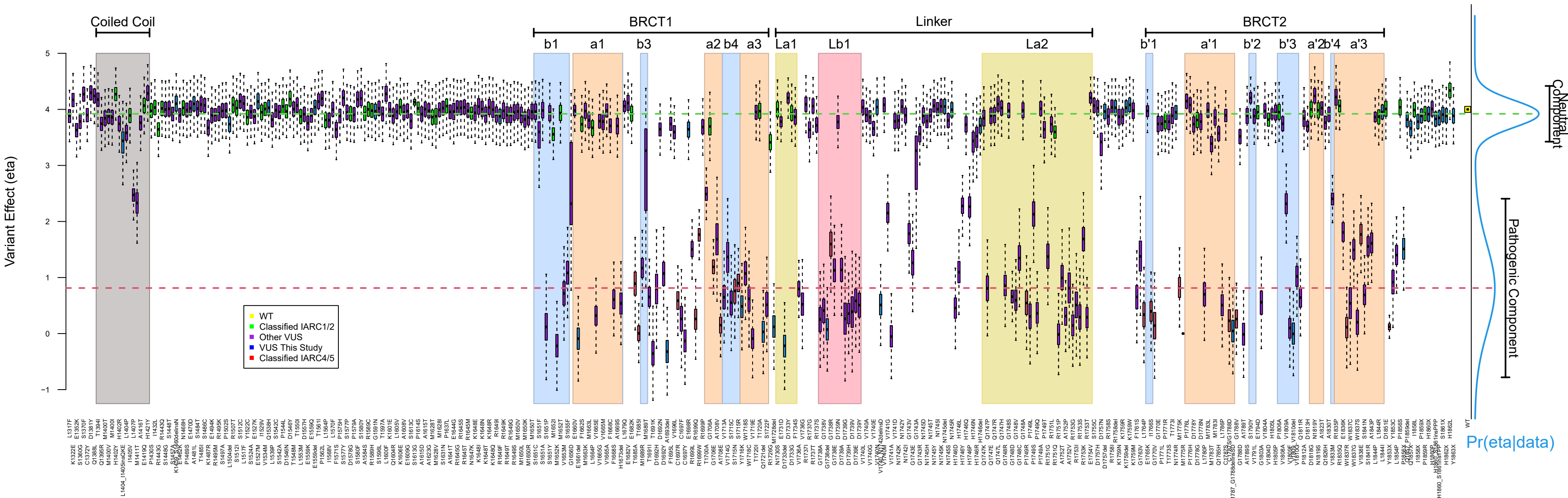

Supplement: Supplementary file 1 — Supplementary Information 1. [file 41598_2022_20500_MOESM1_ESM.pdf]
