# Supplementary material for: Assessment of small in-frame indels and C-terminal nonsense variants of BRCA1 using a validated functional assay
Source: Sci Rep. 2022 Sep 28;12:16203. doi: 10.1038/s41598-022-20500-4 (PMC9519549; doi:10.1038/s41598-022-20500-4)
Supplement: Supplementary file 2 — Supplementary Information 2. [file 41598_2022_20500_MOESM2_ESM.pdf]

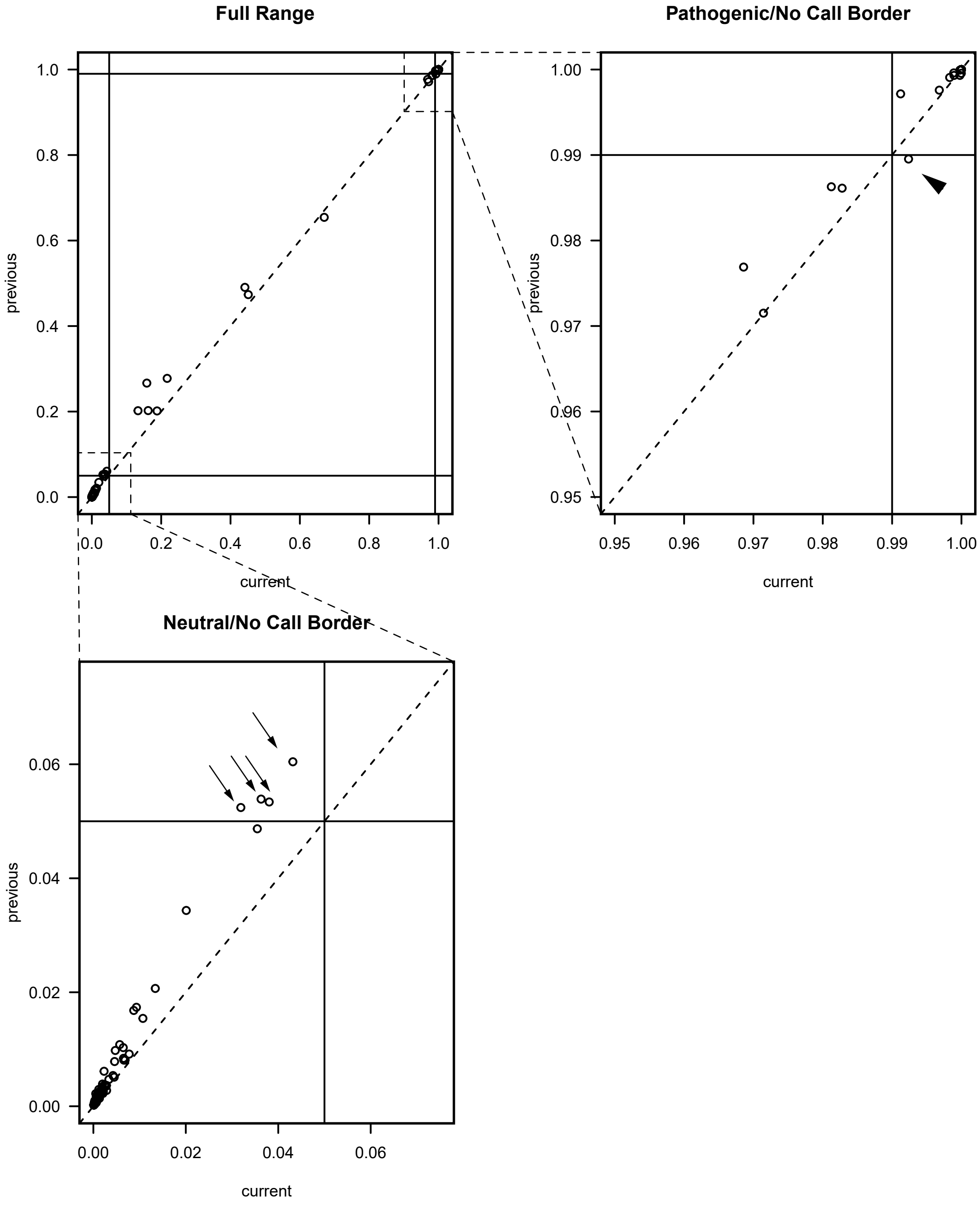

Variant	PrDel	lPostOdds	logBF	PrDel.2018	logBF.2018	lPostOdds.2018
**G1743R**	0.04316573	-3.098583	-2.285249	0.06043956	-1.885434	-2.743768
**P1749T**	0.03804391	-3.230228	-2.416893	0.05338645	-2.016999	-2.875334
**G1788D**	0.03628669	-3.279343	-2.466008	0.05388988	-2.007082	-2.865416
**A1708V**	0.99237102	4.868144	5.681478	0.98953233	5.407276	4.548941
**L1404P**	0.03190728	-3.412494	-2.599159	0.05241329	-2.036424	-2.894758

Supplementary Figure S2. Plot showing the high correlation of the posterior odds in the currently analysis with the posterior odds in previous analysis. Note that when compared with a previous VarCall analysis, four variants moved from a no-call range (VUS) to neutral/benign (arrows), and one variant moved from a no-call region to pathogenic (arrowheads).
