# Supplementary material for: Assessment of small in-frame indels and C-terminal nonsense variants of BRCA1 using a validated functional assay
Source: Sci Rep. 2022 Sep 28;12:16203. doi: 10.1038/s41598-022-20500-4 (PMC9519549; doi:10.1038/s41598-022-20500-4)
Supplement: Supplementary file 3 — Supplementary Information 3. [file 41598_2022_20500_MOESM3_ESM.pdf]

Original images for blots

Figure 1C left panel

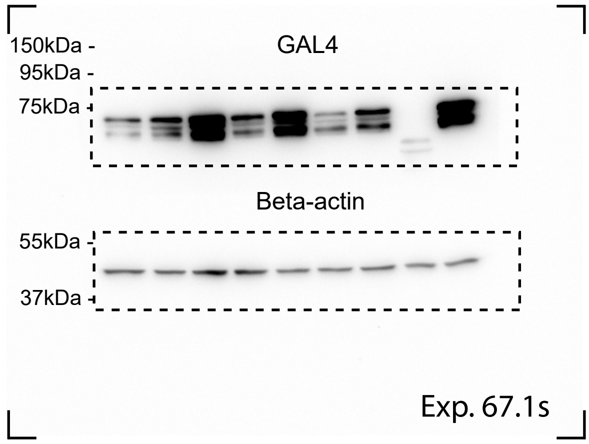

Figure 1C right panel

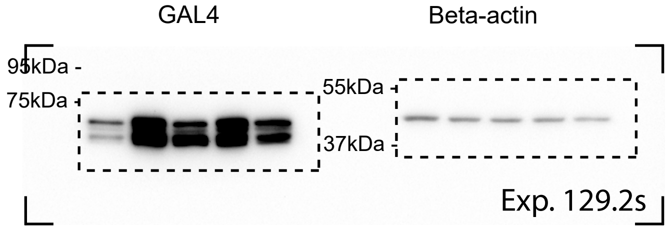

Figure 2C left panel

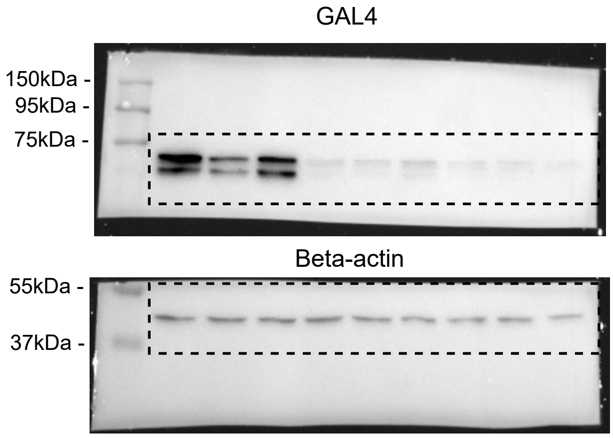

Figure 2C right panel

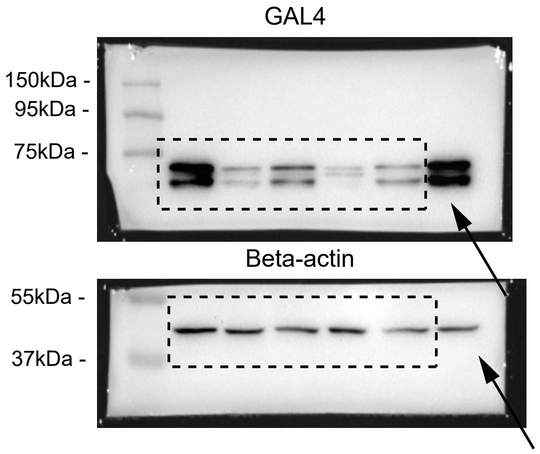

Figure 3C

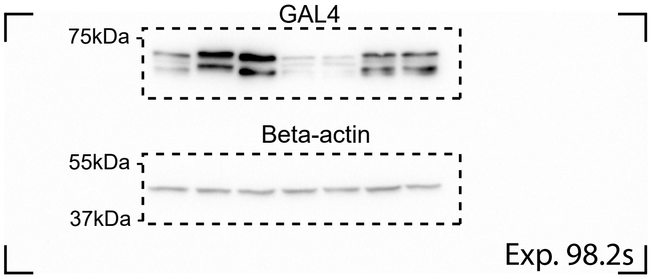

Figure 4E left panel

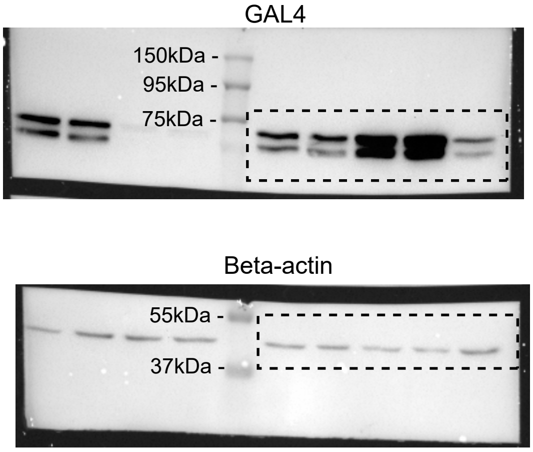

Figure 4E right panel

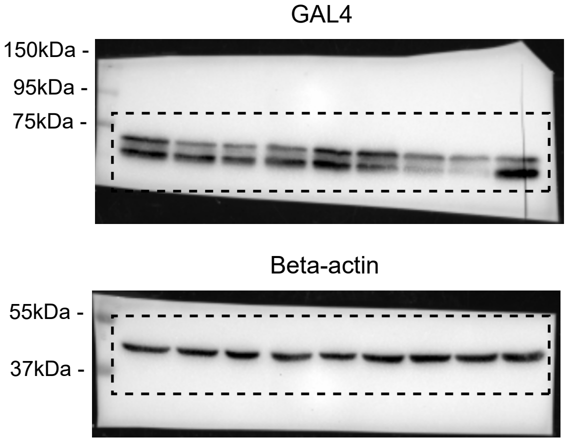

Supplementary Figure S3. Original images of immune blots captured using Bio-Rad ChemiDoc MP imaging system and image acquisition was optimized for the wild-type level of expression using the high-resolution default of the Bio-Rad Image Lab software. Dashed lines indicate where the images were cropped to be shown in Figures 1-4. Irrelevant lanes removed from final figure are indicated with arrow.
